# To bite or not to bite! A questionnaire-based survey assessing why some people are bitten more than others by midges

**DOI:** 10.1186/1471-2458-10-275

**Published:** 2010-05-25

**Authors:** James G Logan, James I Cook, Nina M Stanczyk, Emma NI Weeks, Sue J Welham, A Jennifer Mordue(Luntz)

**Affiliations:** 1 Centre for Sustainable Pest and Disease Management, Rothamsted Research, Harpenden, Hertfordshire AL5 2JQ, UK; 2 Centre for Mathematical and Computational Biology, Rothamsted Research, Harpenden, Hertfordshire AL5 2JQ, UK; 3 School of Biological Sciences, University of Aberdeen, Tillydrone Avenue, Aberdeen AB24 2TZ, UK

## Abstract

**Background:**

The Scottish biting midge, *Culicoides impunctatus*, responsible for more than 90% of biting attacks on human beings in Scotland, is known to demonstrate a preference for certain human hosts over others.

**Methods:**

In this study we used a questionnaire-based survey to assess the association between people's perception of how badly they get bitten by midges and their demographic, lifestyle and health related characteristics.

**Results:**

Most people (85.8%) reported being bitten sometimes, often or always with only 14.2% reporting never being bitten by midges when in Scotland. There was no association between level of bites received and age, smoking, diet, exercise, medication, eating strongly flavoured foods or alcohol consumption. However, there was a strong association between the probability of being bitten and increasing height (in men) and BMI (in women). A large proportion of participants (33.8%) reported experiencing a bad/severe reaction to midge bites while 53.1% reported a minor reaction and 13.1% no reaction at all. Also, women tend to react more than men to midge bites. Additionally, the results indicated that the susceptibility to being bitten by midges is hereditary.

**Conclusions:**

This study suggests that midges prefer to bite men that are tall and women that have a large BMI, and that the tendency for a child to be bitten or not could be inherited from their parent. The study is questionnaire-based; therefore, the interpretation of the results may be limited by the subjectivity of the answers given by the respondents. Although the results are relevant only to the Scottish biting midge, the approach used here could be useful for investigating human-insect interactions for other insects, particularly those which transmit pathogens that cause disease.

## Background

The Scottish biting midge, *Culicoides impunctatus*, is responsible for more than 90% of biting attacks on human beings in Scotland. The painful reactions caused by the bites discourage tourists and disrupt outdoor industries including agriculture and forestry, causing significant losses to the British economy [1]. An estimated 20% loss in working hours each year has been reported in the forestry industry due to midge bites alone [2]. In extreme circumstances, a rate of more than 40 000 midges landing on one forearm every hour has been recorded [3].

Biting midges are known to demonstrate a preference for certain human hosts over others. Anecdotal evidence relating to this phenomenon is now supported by recent scientific evidence. We have shown that host preference is controlled by differences in body odour profiles of different individuals, with non-attractive people producing natural "repellents" [4]. Although the chemical basis for differential attraction to midges is now known, we do not know whether there are particular characteristics or lifestyles that make someone more or less susceptible to attack by midges.

The body odours of many vertebrates (including human beings) are known to provide information about individual identity, sex, reproductive status and health to other members of the same species [5]. Therefore, odours that are specifically associated with such factors could influence biting insects during the selection of an appropriate human host. This has never been investigated for biting midges, but some evidence exists in studies with mosquitoes. For example, some studies have shown that species of *Anopheles *(malaria mosquitoes) preferentially feed on adults rather than children [6-12]. Similarly, Michael *et al*. [13] demonstrated that the biting rates of *Culex quinquefasciatus *(the Southern house mosquito) had a positive association with age in children. It has also been shown that men are bitten more readily than women [10,14] and that larger people are also bitten more often [11]. Additionally, it has been shown that women are more likely to be bitten when pregnant [15]. Other conflicting studies have indicated that certain mosquito species will feed randomly on individuals regardless of age, sex or size [11,14,16]. There is also evidence that the level of attractiveness to mosquitoes could be hereditary. For example, Kirk *et al*. [17] performed a survey on adolescent twins and demonstrated that there was a strong genetic influence on frequency of being bitten by mosquitoes in children between the ages of 12 and 14.

Although there is anecdotal evidence for the association between certain lifestyle, health and demographic characteristics and level of attractiveness to midges, this has never been examined in detail. The aim of this study was to use, for the first time, a questionnaire-based survey to investigate whether the above factors have any association with attraction to midges and reaction to midge bites.

## Methods

The survey was completed by volunteers who were participants or spectators at a duathlon event (First Monster Challenge) held in Inverness, Scotland in September 2008. The cohort involved was thus fairly uniform with the majority of respondents being young, fit and healthy adults. All questionnaires were either distributed the evening before the event and collected the following day or handed out and collected on the day of the event.

A structured self administered questionnaire survey, using both closed and open-ended questions, was developed to investigate details about the level of attractiveness to midges in Scotland and how this relates to other demographic, lifestyle and genetic factors. The questionnaire consisted of 28 questions in five sections: (1) demographics (including age, sex, height, weight and place of residence); (2) lifestyle/health; (3) perceived level of attractiveness to midges; (4) perceived level of attractiveness of their offspring (expressed as first child) to midges; (5) reproductive state (women only). For perceived level of attractiveness to midges, volunteers were asked "when in Scotland, in an area with midges, how often do you get bitten, when on your own?" and were given answer options of "never", "sometimes", "often" or "always". All other factors relating to demographics, lifestyle, etc were cross referenced to this question. Volunteers were also asked "when in Scotland, in an area with midges, how often do you get bitten, when you are with others?". The answers to this question were highly correlated with the answers to the question referring to when the volunteers were on their own, therefore, we have not included these results.

Participants were also asked "when midges bite how do you react?" and were given four options "no reaction" (no mark on skin), "minor" (raised red mark that disappears quickly), "bad" (raised red itchy mark that lasts for a few days) and "very bad" (large red mark with blisters giving pain and itching). Again, factors relating to demographics and lifestyle, etc were cross referenced to this question. The study was approved by the North of Scotland Research Ethics Committee (Ref 07/S0801/51).

### Statistical analysis

Data for level of attractiveness (*i.e*. "never", "sometimes", "often" and "always") were combined to give two categories "never bitten" (category "never" only) and "bitten" (a combination of categories "sometimes", "often" and "always"). Data for reaction to bites ("no reaction", "minor", "bad" and "very bad") were combined to give three categories "no reaction", "minor" and "bad" (a combination of "bad" and "very bad"). Descriptive statistics were then performed on the data using GenStat (version 12.1.0.3412). Two-way cross tabulations were used to analyse associations between level of attractiveness and the following variables: age, sex, height, weight, BMI, place of residence, smoking habit, diet, allergies, exercise, medication intake (including oral contraceptives), alcohol consumption and menstrual cycle stage (women only). For this purpose, the continuous variables age, height, weight and BMI were grouped to form categorical variables. In these cases, category boundaries were chosen to be both easily interpretable and to give a reasonable spread of participants across categories. To determine whether susceptibility to being bitten by midges can be inherited, a cross tabulation was also done to investigate associations between level of attractiveness of parents and their children. A chi-square test of independence was done on all two-way tables and, due to small sample sizes for certain categories, *p*-values were generated from a permutation test using the method of Roff & Bentzen [18]. When the chi-square test indicated association between the variables, further models were fitted. Where one of the variables could be construed as a binary response, logistic regression was used to identify the categories where the response differed. Where all variables had more than two categories, multinomial models were fitted, and residuals from a model of conditional independence were used to identify categories contributing to the association.

For the variables height, weight and BMI, where the distribution differed markedly between men and women, three-way tabulations were formed for the level of attractiveness ("never bitten" vs. "bitten") against both sexes and height, weight or BMI. Logistic regression was used to analyse these data, using height, weight or BMI as a continuous variable.

## Results

### Participant summary

A total of 325 participants took part in this survey. The majority (66.2%) were male and most were within the 'under 31' or '31-40' age categories (30% and 33% respectively) and there was an equal age distribution across both sexes (*p *= 0.46). The majority of participants were fit and healthy with most people (76.9%) doing more than 3 hours of exercise per week and only 6.6% doing less than 1 hour per week. Forty five percent of males and 53% of females had a normal BMI (between 21 and 30). Most people lived in Scotland (71%) with the majority of these living in the west of the country (Table 1).

**Table 1:** Characteristics of the respondents

		Male		Female		Total	
Age (n = 325)	*Under 31*	38	(34.5)	62	(28.8)	100	(30.8)
	*31-40*	31	(28.2)	79	(36.7)	110	(33.8)
	*41-50*	30	(27.3)	53	(24.7)	83	(25.5)
	*Over 50*	11	(10.0)	21	(9.8)	32	(9.8)
							
BMI (n = 307)	*<21*	22	(10.6)	30	(30.3)	52	(16.9)
	*21-23*	46	(22.1)	24	(24.2)	70	(22.8)
	*23-25*	65	(31.3)	21	(21.2)	86	(28.0)
	*25-27*	44	(21.2)	11	(11.1)	55	(17.9)
	*>27*	31	(14.9)	13	(13.1)	44	(14.3)
							
Height (cm) (n = 318)	*<165*	3	(1.4)	41	(38.3)	44	(13.8)
	*165-175*	38	(18.0)	57	(53.3)	95	(29.9)
	*175-185*	119	(56.4)	8	(7.5)	127	(39.9)
	*>185*	51	(24.2)	1	(0.9)	52	(16.4)
							
Weight (kg) (n = 311)	*<60*	4	(1.9)	36	(35.6)	40	(12.9)
	*60-70*	35	(16.7)	47	(46.5)	82	(26.4)
	*70-80*	84	(40.0)	13	(12.9)	97	(31.2)
	*>80*	87	(41.4)	5	(5.0)	92	(29.6)
							
Exercise (n = 316) (per week)	*<1 hour*	9	(4.3)	12	(11.0)	21	(6.6)
	*1-2 hours*	31	(15.0)	21	(19.3)	52	(16.5)
	*3-4 hours*	71	(34.3)	44	(40.4)	115	(36.4)
	*>4 hours*	96	(46.4)	32	(29.4)	128	(40.5)
							
Smoking (n = 318)	*No*	198	(94.3)	93	(86.1)	291	(91.5)
	*Yes*	12	(5.7)	15	(13.9)	12	(8.5)
							
Illness (n = 306)	*No*	193	(97.0)	96	(89.7)	289	(94.4)
	*Yes*	6	(3.0)	11	(10.3)	17	(5.6)
							
Dietary specification (n = 320)	*None*	192	(91.0)	100	(91.7)	292	(91.2)
	*Other*	19	(9.0)	9	(8.3)	28	(8.8)
							
Strongly flavoured food (per week) (n = 313)	*<1 time*	38	(18.5)	25	(23.1)	63	(20.1)
	*1-3 times*	116	(56.6)	53	(49.1)	169	(54.0)
	*>3 times*	51	(24.9)	30	(27.8)	81	(25.9)
							
Alcohol units (per week) (n = 316)	*0*	24	(11.6)	26	(23.9)	50	(15.8)
	*1-5*	94	(45.4)	47	(43.1)	141	(44.6)
	*6-10*	52	(25.1)	27	(24.8)	79	(25.0)
	*>10*	37	(17.9)	9	(8.3)	46	(14.5)
							
Oral contraceptives (n = 95)	*No*	-		75	(78.9)	-	
	*Yes*	-		20	(21.1)	-	
							
Menstrual cycle (n = 54)	*Menstruating*	-		10	(18.5)	-	
	*<15 days*	-		32	(59.3)	-	
	*16-30 days*	-		12	(22.2)	-	
							
Residence (n = 305)	*England*	21	(7)	67	(22)	88	(29)
	*E Scotland*	13	(3.9)	25	(8.1)	38	(12)
	*SE Scotland*	5	(2)	30	(10)	35	(12)
	*SW Scotland*	13	(4.2)	30	(9.8)	43	(14)
	*W Scotland*	55	(17.9)	46	(15.1)	101	(33)
Total		215	(66.2)	110	(33.8)	325	(100.0)

Values given are numbers (percentages) of respondents

### Level of being bitten

When the participants were asked the question "when in Scotland alone in an area with midges, how often are you bitten?" the majority (85.8%, n = 310) said they were bitten sometimes, often or always with only 14.2% claiming to never be bitten. When the participants were asked the question "when in Scotland in an area with midges with others, how often are you bitten?" the majority (88.4%, n = 303) said they were bitten sometimes, often or always with only 11.6% claiming to never be bitten. There was a very high association (*p *< 0.001) between the two questions, with only 9.2% (28 out of 303) participants giving different answers to the two questions.

The data for other factors were then cross-referenced with the answers to the question "when in Scotland alone in an area with midges, how often are you bitten?". Because of possible variation in people's ability to accurately recall, we compared this with their actual perceived level of being bitten on the day of the survey when there were midges present and people were being bitten. There was a strong positive association between the level of being bitten usually compared with level of being bitten on that day (*p *< 0.001).

There was a strong association between where people live and how much they report they are usually bitten (*p *= 0.004). The probability of being bitten was significantly greater for people living in west Scotland and significantly less for those living in England (Table 2).

**Table 2:** Predicted probability of being bitten when in Scotland classified by residence

	Probability	Standard Error
England	0.74	0.049
East Scotland	0.92	0.046
SE Scotland	0.91	0.047
SW Scotland	0.88	0.049
West Scotland	0.94	0.023

Probabilities obtained by logistic regression.

Comparison between regions was done on the logit scale using approximate SEDs

**Figure 1:**
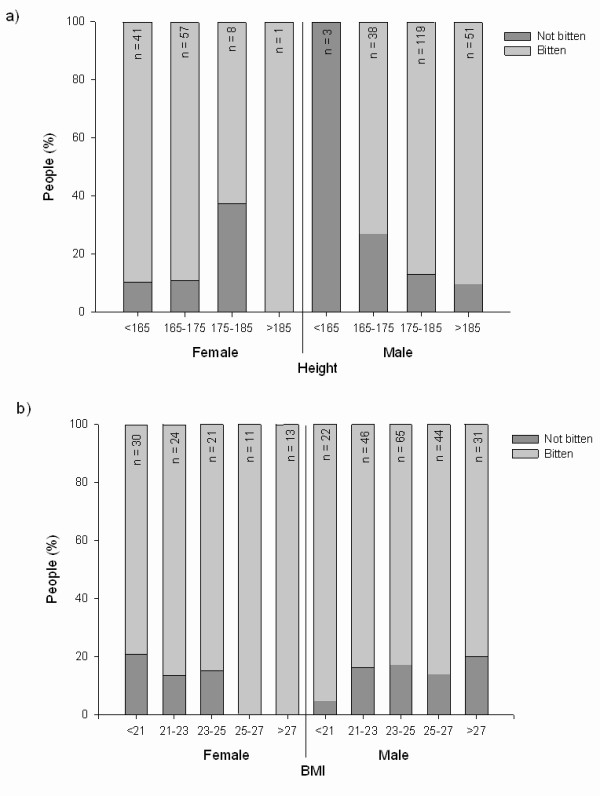
**The number of male and female respondents within different a) height and b) BMI categories in relation to whether they are bitten or not bitten by midges**.

Logistic regression relating the probability of (reporting) being bitten to sex and height showed strong evidence that the probability of being bitten increased with height for men (*p *= 0.006, n = 305; Figure 1) but not for women. The predicted probability of being bitten for men at the 10^th ^(170.7 cm) and 90^th ^(188 cm) sample percentiles of height distribution are 0.7 (SE 0.05) and 0.92 (SE 0.26). Weight showed no relationship to the probability of being bitten (*p *= 0.79, n = 300). However, there was evidence that the probability of being bitten increased with BMI for women (*p *= 0.050, n = 296) but not for men. The predicted probability of usually being bitten in Scotland for females at 10^th ^(19.42) and 90^th ^(27.90) sample percentiles of BMI distribution are 0.77 (SE 0.072) and 0.96 (SE 0.02) respectively (Figure 1).

**Figure 2:**
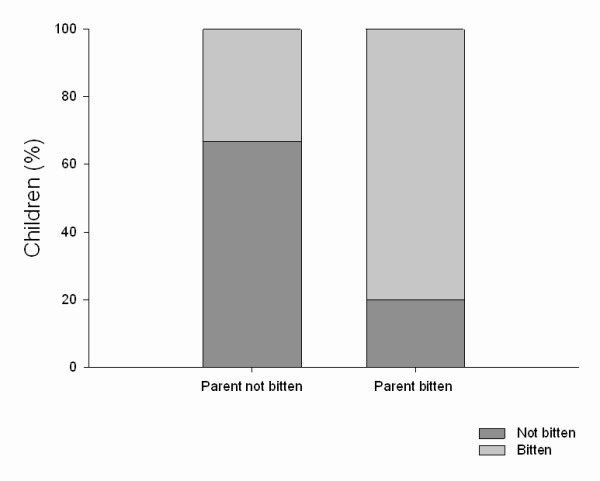
**Percentage of children bitten and not bitten by midges depending on whether their parents are bitten or not bitten**.

There was a strong interaction between the frequency of bites the first child receives and how often the parent is usually bitten (*p *< 0.001, n = 146). Logistic regression showed that the probability of the first child not being bitten by midges was much higher if the parent also claimed to be usually not bitten (*p *< 0.001). There was no evidence that the sex of the parent had an effect on the rate of biting on the child (*p *= 0.11) (Figure 2).

There was insufficient variation in the responses to reliably assess association between ethnic origin, general diet, medication or illness and whether people are bitten or not bitten by midges. There was no significant association between age, sex, smoking, exercise, eating strongly flavoured foods (*e.g*. garlic, chilli and onions), frequency and amount of alcohol consumption and whether people are bitten or not bitten by midges (*p *> 0.05). In women there was no evidence for an interaction between level of biting and the reproductive status (*i.e*. premenopausal, menopausal or postmenopausal) (*p *= 0.99, n = 62), whether the respondents were taking oral contraceptives (*p *= 0.44, n = 91), or the stage of the menstrual cycle (*p *= 1.00, n = 51). There was some evidence of a significant association with allergy to insect bites and being bitten (*p *= 0.05, n = 310), due to a high proportion of people with insect allergy reporting that they are "always bitten" when in Scotland. This association disappears (*p *= 0.99) for the binary (not bitten vs. bitten) response.

### Level of reaction to bites

For participants' reported reactions to midge bites, data in the "bad" and "very bad" categories were combined in the analysis because there were only 11 responses in the "very bad" category (3.6%, n = 305). The majority of people (53.1%) reported a minor reaction and 33.8% reported a bad reaction. Only 13.1% claimed that they have no reaction to midge bites.

**Figure 3:**
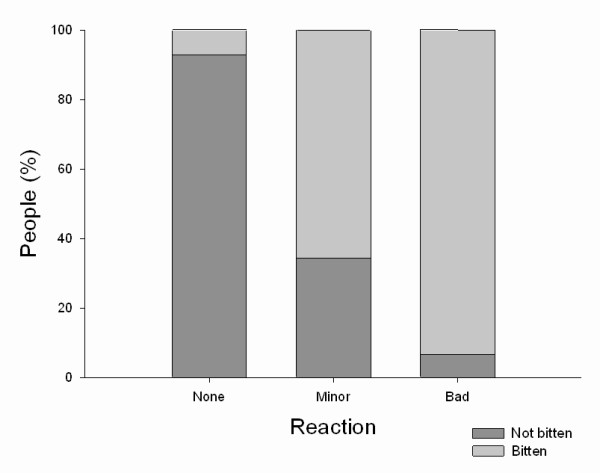
**Reaction to midge bites and level of being bitten**.

There was a strong association between the frequency of being bitten and the reaction to bites with most people who report reacting badly to bites also receiving high numbers of bites and with those who do not react badly claiming they never get bitten (*p *< 0.001, Figure 3).

**Figure 4:**
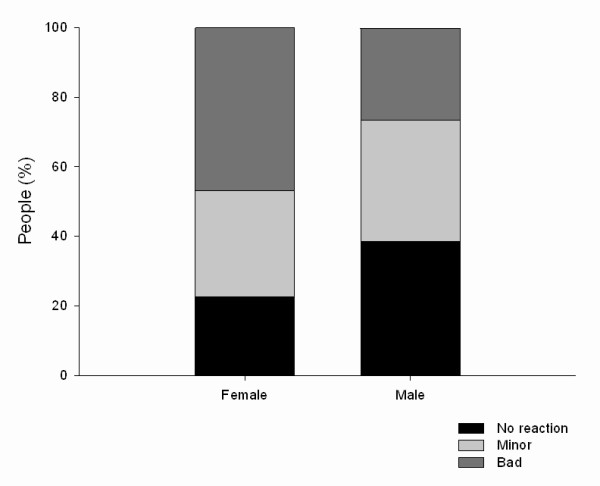
**Reaction of women and men to midge bites**.

There was no correlation between age and reaction to bites (*p *= 0.46, n = 305). However, there was a significant association of reaction to bites in relation to sex (*p *= 0.005), which was caused by women having a significantly higher probability of reporting a bad reaction to midge bites (*p *= 0.01) (Figure 4).

There was no association between residence (*p *= 0.37, n = 292), eating strongly flavoured foods (*e.g*. chilli, garlic, onions, *p* = 0.14, n = 300) or the frequency (*p *= 0.70, n = 301) and amount (*p *= 0.36, n = 303) of alcohol ingestion, reproductive status (*p *= 0.18, n = 61), contraceptive usage (*p *= 0.86, n = 90) or menstrual stage (*p *= 0.94, n = 52) and reaction to midge-bites. Neither smoking (*p *= 0.27, n = 304) or allergies (including allergies to animals, antibiotics, food, hay fever and pollen) had any effect on reaction to bites either amalgamated (*p *= 0.98, n = 304) or individually. However, there was a significant association between people reporting an allergy to insects and their reaction to bites, with people with an insect allergy reacting more badly to bites than those without (*p *= 0.03, n = 305), as would be expected.

There was also a significant association between exercise and reaction to midge bites (*p *= 0.04, n = 303). A multinomial model indicated that people doing the least exercise (less than one hour per week) and most exercise (more than 4 hours) were more likely to have no reaction to midge bites than people who do between 1 and 4 hours of exercise per week. Logistic regression showed no significant interaction between level of reaction and height (*p *= 0.59, n = 300), weight (*p *= 0.74, n = 296) or BMI (*p *= 0.18, n = 292) (when differences due to sex had been accounted for).

## Discussion

Despite the vast amount of anecdotal evidence surrounding why some people get bitten more than others by blood-feeding insects, few studies have investigated the demographic, lifestyle and health factors involved. This is the largest investigation of its type for any biting insect. Our finding that only 14.2% of people claim to never be bitten is the first measurement of differential attraction to biting insects using a large sample size. To our knowledge this is the first reported statistic of the prevalence of the trait for being "unattractive" to biting insects and supports our previous work on mosquitoes and midges on much smaller sample sizes when directly measuring attractiveness and odour profiles of individuals [4,19].

It is well known that the west coast of Scotland is an area of high midge populations and, therefore, it was expected to find that people who claim they are bitten at home the most indeed lived in the west of Scotland. This geographic region provides a unique environment which allows the Scottish biting midge to thrive successfully [3].

Our study is the first to show an association between body size and attractiveness to biting midges. The preference for biting people of a greater height could be associated with midge behaviour and flight patterns. For example, Venter *et al*. [20] demonstrated, using light traps, that most midges are caught at a height of 2.8 metres above ground level. Midges are also known to rest in trees after they have emerged from pupae and are found in greater numbers with increasing height (in particular from 1-4 metres) [21]. It is, therefore, possible that midges searching for a suitable host would be descending from above and would encounter taller people, within in a group, first. Additionally, larger people would provide a more substantial visual target for host-seeking midges as well as greater amounts of heat, moisture and attractant semiochemicals (behaviour modifying chemicals), such as carbon dioxide, which are all involved in the location of a suitable blood meal. These findings are also supported by similar studies with mosquitoes. For example, Port *et al*. [11] demonstrated that larger people are bitten more often by certain mosquito species. Similarly, men have been shown to be bitten more readily than women in previous studies, however, whether this is due to true differences between the sexes (a pattern not found here) or due to differences in body size is unknown [10,14,22].

Some investigations have provided evidence that certain mosquito species will preferentially feed on adults rather than children [6-12]. Similarly, Michael *et al*. [13] demonstrated that biting rates of *Culex *species mosquitoes had a positive association with age in children. In this study, the participants were adults so we could not assess whether there is a difference between children and adults. However, we did not find any association of age with the level of being bitten by midges. There is a reported link between alcohol ingestion and attractiveness to biting insects suggesting that people who drink alcohol are more attractive to mosquitoes [23,24]. Anecdotal evidence for this certainly exists in relation to midges, however, in our study we found no such association. There was also no association between being bitten and the consumption of strongly flavoured foods (*e.g*. garlic, chilli and onions). This is contrary to popular belief as people commonly believe that garlic, in particular, makes you less attractive to biting insects. In support of our findings, Rajan *et al*. [25] found no effect of garlic consumption on how many mosquito bites were received in laboratory trials. There is also much speculation that other factors such as smoking, exercise, diet and reproductive stage can be related to whether people are bitten or not bitten by midges [26]. However, no such associations were found in this study. This suggests that these factors do not influence the cues used by midges during their search for a potential blood meal.

The interaction between the frequency of bites the first child receives and how often the parent is usually bitten suggests that the factor that makes someone attractive or unattractive to midges may be hereditary and could be controlled genetically. Kirk *et al*. [17] performed a survey on adolescent twins and also suggested that there was a strong genetic influence on frequency of being bitten by mosquitoes of children of certain ages. Other studies have shown that odours of twins are similar and thus may indicate the importance of genetic makeup on body odour [27]. Logan *et al*. [4,19] demonstrated that the reason some people are bitten less by mosquitoes and midges is due to the production of repellent chemicals in their body odour. Therefore, it is plausible that the biosynthesis or release of such natural repellents is under genetic control and is hereditary [26]. Although differences in parental awareness of bites received by their children may also play a role here, there appears to be growing evidence that the level of attractiveness to biting insects is under genetic control. We are currently investigating further the possible genetic control of attractiveness to biting insects in molecular studies in our laboratories.

For the first time we also report the proportion of people who claim to react differently to bites from midges and our results suggest that, despite the immense annoyance factor caused by these insects, the majority of people sustain a minor reaction whilst 33% report having a bad reaction. Those that report having no reaction to midge bites are in the minority with a strong association between the frequency of being bitten and the reaction to bites. This may be a direct association with number of bites received (*e.g*. the more bites received, the higher the level of perceived reaction) or it may indicate that there is an indirect link between level of attractiveness and level of reaction, that does not relate to the number of bites received. Indeed the severity of cutaneous reactions to insect bites is known to differ between individuals and, therefore, there could be an association [28,29]. Additionally, other studies have shown that people's perception of how attractive they are (in a questionnaire) matches well with their accurately measured level of attractiveness to mosquitoes in the laboratory [4,19,26]. However, no study has ever investigated this link and we cannot make definitive conclusions from our own study. Nevertheless, it does suggest that people's perception of how attractive they are to mosquitoes could be influenced by their reaction to the bites they receive rather than the number of bites. In support of this, Read *et al*. and Peng *et al*. [30,31] also demonstrated a lower than expected correlation between reported bites of volunteers and the number of mosquitoes caught in traps using their body odours in a public perception of mosquito annoyance survey.

In our study we found a significant association of reaction to midge bites in relation to sex, which was seen in women having a significantly higher probability of a bad reaction to midge bites than men. Women may experience a greater reaction to midge bites or may have a greater awareness of bites received. Further sensitivity studies would need to be done to confirm this result, although other studies have also suggested, by measuring the level of mosquito saliva-specific antibodies in infants, that there may be similar differences between the sexes in terms of their reaction to bites from insects [32].

The study is not without limitations as our respondents were participants, spectators and organisers of a duathlon event and consequently the majority of respondents were young, fit and healthy. Additionally, it relied on subjective answers from volunteers rather than experimental measurement of level of attractiveness of the participants. However, measurement of every individual would have been logistically impossible. The data have been analysed using the answers to the questions "how often are you usually bitten when in Scotland?". Because of possible variation in people's ability to accurately remember, we compared this with their perceived level of being bitten on the day of the survey when there when people were being bitten and we found a strong positive association. We also have evidence from previous investigations that the perceived level of being bitten by biting insects is directly correlated to their actual level of "attractiveness" when measured accurately using a laboratory-based behavioural experiment with mosquitoes. Additionally, we have previously demonstrated that the level of attractiveness to midges is reflected in the response of mosquitoes to the same people, indicating that the results of this study could be extrapolated to instances where people are bitten by mosquitoes, including those that vector pathogens [4,19].

## Conclusions

In summary, we found that 14% of people do not attract midges and only a small proportion report no reaction to midge bites. This study suggests that midges prefer to bite men that are tall and women that have a large BMI, and that the tendency for a child to be bitten or not could be inherited from their parent. Also, women tend to react more than men to midge bites. Contrary to common belief diet, health and other lifestyle factors do not play a role in making people more or less attractive to midges. The study is questionnaire-based; therefore, the interpretation of the results may be limited by the subjectivity of the answers given by the respondents. Although the results are relevant only to the Scottish biting midge, the approach used here could be useful for investigating human-insect interactions for other insects, particularly those which transmit pathogens.

## Competing interests

The authors declare that they have no competing interests.

## Authors' contributions

JGL, AJM, NMS, ENIW and JIC designed the research question. JGL, SJW and ENIW conducted the analysis and drafted the manuscript. JGL wrote the final manuscript. AJM, NMS, ENIW, JIC and SJW participated in writing the manuscript. All authors have read and approved the final manuscript.

## Pre-publication history

The pre-publication history for this paper can be accessed here:

http://www.biomedcentral.com/1471-2458/10/275/prepub
